# Improved multi-objective clustering algorithm using particle swarm optimization

**DOI:** 10.1371/journal.pone.0188815

**Published:** 2017-12-05

**Authors:** Congcong Gong, Haisong Chen, Weixiong He, Zhanliang Zhang

**Affiliations:** PLA University of Science and Technology, Nanjing, PR China; Beihang University, CHINA

## Abstract

Multi-objective clustering has received widespread attention recently, as it can obtain more accurate and reasonable solution. In this paper, an improved multi-objective clustering framework using particle swarm optimization (IMCPSO) is proposed. Firstly, a novel particle representation for clustering problem is designed to help PSO search clustering solutions in continuous space. Secondly, the distribution of Pareto set is analyzed. The analysis results are applied to the leader selection strategy, and make algorithm avoid trapping in local optimum. Moreover, a clustering solution-improved method is proposed, which can increase the efficiency in searching clustering solution greatly. In the experiments, 28 datasets are used and nine state-of-the-art clustering algorithms are compared, the proposed method is superior to other approaches in the evaluation index ARI.

## Introduction

Knowledge discovery is a process of analyzing data from different prospective and summarizing it into useful information [1]. These methods include a number of technical approaches, such as classification, data summarization, regression and clustering [2]. Technically, clustering is a process of grouping the data instances based on one or more features of the data[3]. As a popular data analysis technique, clustering has attracted particular attention from researchers in recent years because of the need to analyze and understand information hidden in the datasets coming from different sources. The applications of cluster analysis have been used in a wide range of different areas such as face recognition [4], spatial database analysis [5] and traffic incidents [6].

The most popular algorithms among various clustering techniques are K-means [7] and hierarchical clustering [8,9]. For K-mean algorithm, it creates the partition of data set into *k* number of clusters where *k* is predefined [10]. Hierarchical clustering is a method of cluster analysis which seeks to build a hierarchy of clusters. In the clustering process, those methods focus on obtaining groups by optimizing single objective. However, it is necessary to optimize several objective functions simultaneously in some real-world problems[11].Furthermore, those classical algorithms suffer from disadvantages of initial centroid selection, local optima, low convergence rate [1].

To overcome the drawbacks of these algorithms, PSO-based clustering methods have been studied by the academic community. Particle Swarm Optimization (PSO) is inspired by the social behavior of animals like fish schooling and bird flocking. It has become an efficient method for searching approximate optimal solution due to its simplicity, few parameter configuration and global exploration ability on some complex problems [12]. In addition, PSO is well-suited for multi-objective optimization as their use of a population enables the whole Pareto set to be approximated in a single algorithm run [3].

In the early years, researchers use PSO to perform single-objective optimization on clustering problems. An implementation of PSO for data clustering was introduced in [13]. In the algorithm, standard *gbest* PSO is used to find the centroids of a specified number of clusters. Shen et al. presented a mountain clustering based on improved particle swarm optimization (MCBIPSO) algorithm [14]. The improved PSO algorithm is used to find all peaks of the mountain function, and the calculation is easier and more efficient in deciding the clustering centers of data samples. Single objective PSO-based methods have used in the document clustering [15], image segmentation [16] and spatial datasets [17].

With the study of the multi-objective PSO algorithms, some of them are introduced to solve the clustering problems. Yang et al. proposed a hybrid clustering algorithm based on PSO and K-harmonic means (PSOKHM) in [18]. It shows that the PSOKHM algorithm increases the convergence speed of PSO, and is also capable of escaping from local optima. Abubaker et al. proposed a new automatic clustering algorithm based on multi-objective PSO and Simulated Annealing (MOPSOSA) in [19]. This method simultaneously optimizes three different objective functions, which are used as cluster validity indexes for finding the proper number of clusters. Armano G et al. combined the combinatorial form of PSO and locus-based adjacency genetic scheme in [20], the algorithm is robust and outperforms in the comprehensive experimental study.

However, previous studies have three limitations. Firstly, clustering problem is a combinatorial problem and the curse of dimensionality may be encountered in large datasets, this will lead to a dramatic deterioration in algorithm performance. Secondly, the distribution of Pareto set in clustering problem has unique feature, which makes the selection of global leader harder. Thirdly, particle representation in previous studies is in discrete space, but the PSO is more suitable for optimization in continuous space[21].

The purpose of this paper is to address the limitations of previous work. Towards this goal, an improved multi-objective clustering framework using particle swarm optimization (IMCPSO) is proposed, which finds well-separated and compact clusters without the predefined number of clusters. Threefold efforts have been made in this paper. Firstly, it attempts to improve the particles during the iterations, which can get better solution with less iteration and alleviate the curse of dimensionality. Secondly, it analyzes the feature of clustering solutions distribution, which helps enhance the performance in leader selection and decision maker. In addition to that, we design a novel particle representation that helps PSO search the clustering solutions in continuous space. It is discovered that IMCPSO is superior to other approaches in terms of accuracy.

The rest of this paper is organized as follows. Section 2 briefly describes the related work about clustering algorithms and multi-objective PSO. Section 3 presents the IMCPSO approach in detail. A comprehensive set of experimental results are provided in Section 4. Finally, Section 5 reports the conclusions of obtained results and suggests some directions of future work.

## Related works

### Clustering algorithms categorized by criterion optimized

Traditional classifications of clustering algorithms primarily distinguish between hierarchical, partitioning, and density-based methods[22,23]. Partitional clustering is dynamic, where data points can move from one cluster to another, and the number of clusters *k* is usually required in advance, e.g. K-means [7]. Hierarchical clustering consists of a sequence of partitions in a hierarchical structure, and takes the form of either agglomerative or divisive method, e.g. average-link hierarchical clustering [8] and Ward method[24]. Density-based methods group dataset into clusters based on density conditions. Clusters of dense regions are separated by regions of low density, such as DBSCAN [25,26], Mean shift[27].

In addition, graph-theoretical technique is used in clustering, e.g. spectral clustering[28] and BIRCH[29]. Since then, AP clustering[30] is proposed and uses the message between data points to determine the center of cluster.

### Multi-objective PSO

In PSO framework, each particle is represented as a potential solution, and the particle achieves global optimization by moving its position in *D*-dimension search space. The velocity ***v***_***i***_ and the position ***x***_***i***_ are updated as follows:

$$v_{i}(t+1)=v_{i}(t)+c_{1}r_{1}({pbest}_{i}-x_{i})+c_{2}r_{2}(gbest-x_{i})$$ (1)

$$x_{i}(t+1)=x_{i}(t)+v_{i}(t)$$ (2)

where ***x***_***i***_ = (*x*_*i*1_,*x*_*i*2_,…,*x*_*iD*_) is the position of the *i*th particle, ***v***_***i***_ = (*v*_*i*1_,*v*_*i*2_,…,*v*_*iD*_) is the velocity of particle *i*, *t* represents the generation number, *c*_1_ and *c*_2_ are local and global learning factors respectively, *r*_1_,*r*_2_ are random numbers between [0, 1], ***pbest***_***i***_ stands as the previous best position for the particle *i*, ***gbest*** is the global best position found so far in the entire swarm. The particle position is updeted by Eq (2).

Added with a inertia weight factor *w*, the algorithm can have a better control over the search scope, and can achieve better results in the application of certain issues[31], and the particle velocity is adjusted to

$$v_{i}(t+1)=w\cdot v_{i}(t)+c_{1}r_{1}(p_{i}-x_{i})+c_{2}r_{2}(g-x_{i})$$ (3)

The first multi-objective particle swarm optimization (MOPSO) is reported by Coello [32]. The MOPSO differs from PSO in that it contains the processes of construction and maintenance of the external archive using the concept of Pareto optimality. Moreover, the mechanism in terms of the selection on ***gbest*** and ***pbest*** is quiet different. Therefore, recent multi-objective PSO works use the concept of Pareto optimality to select non-dominated particles as leaders, in order to make solutions converge to the true Pareto set[33,34].

## Proposed method

In this section, we describe the IMCPSO method in detail. As already pointed out, it is based on the multi-objective PSO algorithm. IMCPSO consists of three main parts: objective functions, optimization and decision-making. Firstly, two conflicting objective functions are defined with the aim of obtaining compact and well-separated clusters. Secondly, we illustrate the process of optimization from three parts: particle representation, selection of leader and solution-improved process, because the optimization is the key work for searching good partitions. Finally, decision maker is used to select the most suitable solution in Pareto set.

### Objective functions

The clustering problem is defined as follows. Consider the dataset *P =* {*p*_1_, *p*_2_, …, *p*_*n*_}, where *p*_*i*_ = (*p*_*i*1_, *p*_*i*2_,…, *p*_*id*_) is a feature vector of *d*-dimensions and also referred to as the object, *p*_*ij*_ is the feature value of object *i* at dimension *j*, and *n* is the number of objects in *P*. The clustering of *P* is the partitioning of *P* into *k* clusters {*C*_1_, *C*_2_,…,*C*_*k*_} with the following properties: (1)${⋃}_{i=1}^{k}C_{i}=P$, (2)*C*_*i*_ ∩ *C*_*j*_ ≠ ∅, such that *i*,*j* ∈ {1,2,⋯,*k*} and *i* ≠ *j*.

Given a clustering solution of the data, numerous measures for estimating its quality exists and the goal is to get most compact and well-separated clusters. Towards this goal, we introduce two objective functions—overall deviation and mean space between clusters—to evaluate the compactness and separability of clusters respectively.

**(a) overall deviation**

The overall deviation of a clustering solution reflects the overall intra-cluster size of the data. The formula is as follows.

$$Dev(C)=\sum_{C_{k}\in C}\sum_{i\in C_{k}}\delta (i,\mu_{k})$$ (4)

where *C* is the set of all clusters, *i* is the element of data, *μ*_*k*_ is the centroid of cluster *C*_*k*_, *δ*(.,.) is the distance function (e.g. Euclidean distance). As an objective, overall deviation should be minimized.

**(b) mean distance between clusters**

This objective function reflects the difference between clusters. It is calculated by the minimum distance of the cluster’s neighbors. In clustering analysis, *neighbor* is a local concept that reflects the relation of two data points. In this paper, we use the Gabriel graph to obtain the adjacency relation of all data points[35]. Gabriel graph is a subgraph of the Delaunay triangulation, which connects two data points *v*_i_ and *v*_j_ for which there is no other point *v*_*k*_ inside the open ball with diameter [*v*_*i*_*v*_*j*_]. The advantage of the Gabriel graph is that it can get the all-connected graphs with appropriate distance[36]. After getting the Gabriel graph of all points, the objective function is calculated as follows.

$$Mdc(C)=\frac{1}{\left|C\right|}\sum_{C_{k}\in C}\left(\min_{i\in C_{k},j\in N_{i},j\notin C_{k},}\delta (i,j)\right)$$ (5)

where *N*_*i*_ is the neighbors set of data *i* in Gabriel graph. As an objective function, *Mdc* should be maximized. In order to minimized the objective as similar as *Dev*, this objective value could be negated (-*Mdc*).

### Particle representation

**Fig 1:**
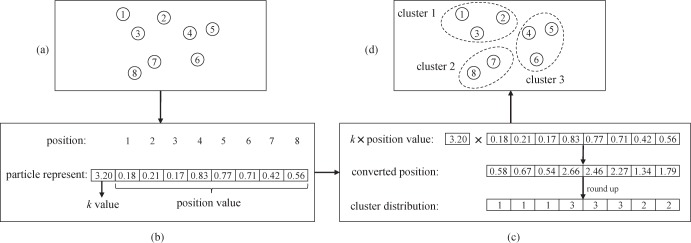
The design of particle representation and conversion of clusters. (a) 8 data points to be clustered. (b) The particle representation, where the vector consisting of nine elements. (c) The process of generating clustering solutions. (d) Clusters according to particle representation.

Generally speaking, the particle representation is important because each particle is represented as a potential solution in PSO framework. Therefore, we design a continuous particle representation for clustering problem. In this scheme, shown in Fig 1, each particle is represented by a vector consisting of *N*+1 elements, where *N* is the number of data points. The first element in particle vector is *k* value and reflects the clusters number of the particle. The *k* value lies in the interval (1,*k*_max_], where *k*_max_ is the maximum number of clusters. The other *N* elements in particle vector are the position values of *N* data points. Each of these elements takes a value in the internal (0,1]. An example of particle representation is shown in Fig 1 (B).

When a particle is converted into a clustering solution, all the position value of *N* data points is multiplied by the *k* value, then round up to obtain a clustering solution. A process of conversion is shown in Fig 1 (C).

**Fig 2:**
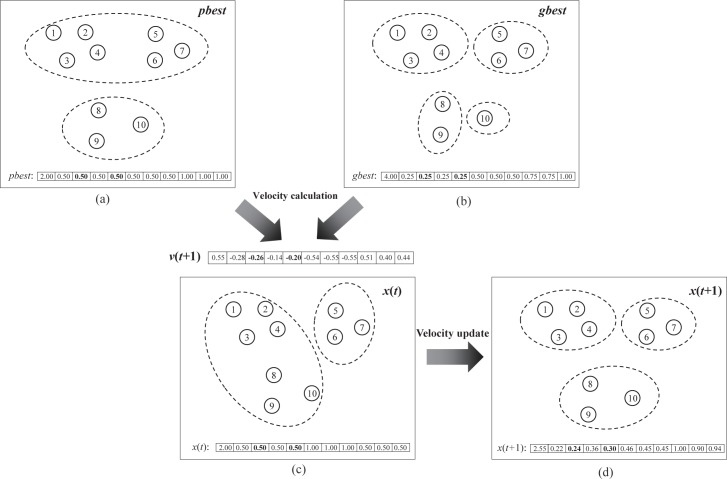
Velocity update process of particles and preserve the similar parts in *pbest* and *gbest*. (a) *pbest* particle. (b) *gbest* particle.(c) particle *x*(*t*) before iteration. (d) particle *x*(*t*) after iteration. The velocity *v*(*t*+1) is calculated by *pbest* and *gbest*. Notice that data 2 and 4 belong to the same cluster in both *pbest* and *gbest*, and the value in *pbest* or *gbest* is the same(0.5 and 0.25 in *pbest* and *gbest*, respectively). The *v*(*t*+1) will make *x*(*t*) move to the same position in the two dimenstion, so that the data 2 and 4 have a high probability of being assigned to the same cluster in the newly generated particle.

The advantages of the proposed particle representation are manifold. On one hand, it can take full advantage of PSO searching in continuous space, since the PSO technique is suitable for finding solutions in continuous non-linear optimization problems [37]. On the other, the partitioning of different number of clusters can be represented, because the k value maintains flexibility to change. In addition, it tends to preserve the similar parts and mutate the different parts of *pbest* and *gbest* when the particle velocity is updated. This feature could help particles find the global optimal solution with inheriting the advantages of the other solutions. An example of iteration in IMCPSO is shown in Fig 2.

### Leader selection strategy

The analogy of PSO with evolutionary algorithms makes evident the notion that using a Pareto optimality scheme could be the straightforward way to extend the approach to handle multi-objective optimization problems[38]. In general, a multi-objective problem of the type:

$$minimize\;f(x)≔[f_{1}(x),f_{2}(x),\cdots ,f_{k}(x)]$$ (6)

subject to

$$g_{i}(x)\leq 0,i=1,2,\cdots ,m$$ (7)

$$h_{i}(x)=0,i=1,2,\cdots ,p$$ (8)

where ***x*** = [*x*_1_,*x*_2_,…,*x*_*D*_]^*T*^ is the vector of decision variables, $f_{i}:R^{n}⟶R,i=1,\ldots ,k$ are the objective functions and $g_{i},h_{j}:R^{D}⟶R,i=1,\ldots ,m,j=1,\ldots ,p$ are the constraint functions of the problem. Given two vectors $x,y\in R^{k}$, we say that ***x* dominates *y*** if *x*_*i*_ ≤ *y*_*i*_ for *i* = 1,…,*k*, otherwise ***x*** is **nondominated** with ***y***. We say that a vector of decision variables is **Pareto-optimal** if it is nondominated with respect to F(F is the feasible region), and the Pareto Optimal Set (or Pareto set) $P^{*}=\{x\in F|x\;\mathrm{is}\;\mathrm{Pareto-optimal}\}$[39].

However, the PSO algorithm with Pareto optimality scheme will produce not only one but a set of nondominated leaders and the selection of an “appropriate” leader becomes difficult, because these nondominated solutions are equally good mathematically. The choosing of *pbest* is relatively simple, which adopts non-dominated particle between current position and previous best position as *pbest*. If they do not dominate each other, then select them randomly[40].

The selection of *gbest* is relatively complicated, as a global leader, the location directly determines the search direction of particles. The main selection of global leader can be categorized as follows: (a) stochastic selection in Pareto set; (b) selection based on crowding density[41]. The former method tends to raise a greater selection probability in regions where particles are concentrated, which is not conducive to the distribution among Pareto set, and drops the diversity of the population. The latter method also tends to lead the particles falls into the local optimum, and the reason will be analyzed next.

#### Analysis of the Pareto set in clustering problem

In the clustering problem, the number of clusters *k* plays an important role in getting good clustering solution. The two objective functions, *Dev* and -*Mdc*, correspond to large *k* values (more compact) and smaller *k* values (well separated) respectively. As a result, different *k* values have relative clear distinction in the Pareto set. The cluster assigned of data points can only be taken in a limited category since the clustering problem is a combinatorial problem. When a particular k^*^ produces a good clustering solution, it will dominate the other solution with the same k^*^, so the Pareto set lies sparse relatively around the k^*^.

**Fig 3:**
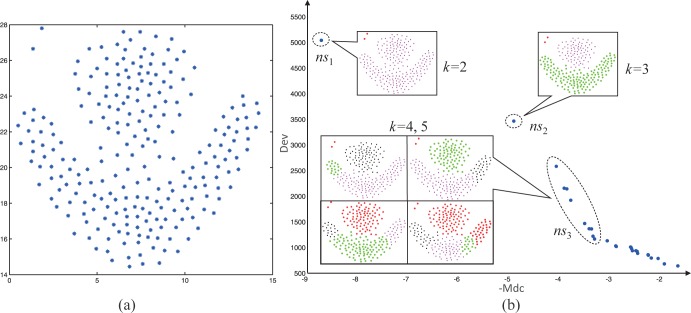
**(a) Data points to be clustered. (b) Pareto set of the clustering solutions, where *k* = 2,3,4,5 from top left to bottom right and *ns* is nondominated solution.** The clustering solutions *ns*_1_ and *ns*_2_ produced by *k* = 2, 3 are better than the other combinations of the same *k*, making the region of pareto set is sparse when k = 2, 3.On the other hand, the clusters are not well separated when *k* = 4,5 and the number of nondominated solutions increases with same *k* (see *ns*_3_).

Fig 3 illustrates Pareto set in a clustering problem, the distributions of the Pareto set would be scattered in the clustering problem (*ns*_1_ and *ns*_1_ in Fig 3). For this reason, the selection based on crowding density is likely to choose a non-dominated solution like *ns*_2_, but no other non-dominated solution is near it, which makes the optimization algorithm falls into local optimum. Furthermore, when the partitions in a better *k*, the clusters are well-separated (*k* = 2,3 in Fig 3) and the corresponding Pareto set is also very sparse. On the contrary, the Pareto set will be dense (*k* = 4,5 in Fig 3) when the *k* is not well-separated.

#### Leader selection strategy of whole process

**Fig 4:**
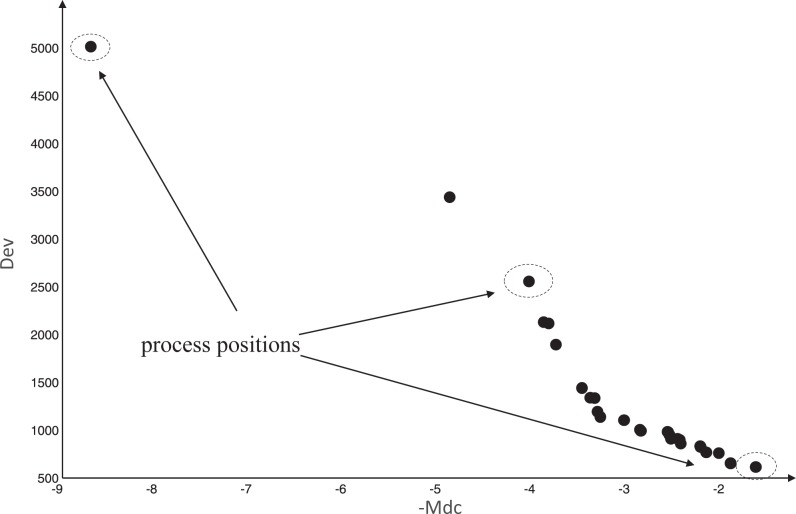
The process particles in a Pareto set.

In order to avoid falling into local optimum, we propose a "leader selection strategy of whole process". The "process particles" we called are one set which contains the positions of the leftmost, middle and rightmost solutions of the Pareto set, as shown in Fig 4. Obviously, the positions of the leftmost and rightmost particles are easily determined. Before the middle particle is determined, all the Pareto solutions are normalized using the following formula.

$$x_{i,j}^{\prime }=\frac{x_{i,j}-min(x_{j})}{\max\left(x_{j}\right)-min(x_{j})}$$ (9)

where *x*_*i*,*j*_ is the value of the *i*th particle on the *j*th objective function, max(*x*_*j*_) and min(*x*_*j*_) are the maximum and minimum of Pareto set on the *j*th objective function, respectively. $x_{i,j}^{\prime }\in [0,1]$ is the objective function value after normalization. The magnitude of each objective function becomes uniform and the fitness of each Pareto solution can be obtained by summing each objective function, as shown in the following formula, where *m* is the number of objective functions.

$${fit}_{i}=\sum_{j=1}^{m}x_{i,j}^{\prime }$$ (10)

Thus, the selection of middle particle is the Pareto solution with the minimum *fit*_*i*_. Obviously, the middle particle is a good trade-off solution of all objective function. After the process particles are determined, the global leader is one of them, where selection probability of middle particle should be higher (0.7, empirically), and the leftmost and rightmost particles are assigned a lower probability (both 0.15, empirically).

The advantages of leader selection strategy of whole process are: (a) it can lead particles to traverse all possible Pareto sets, and is not easy to fall into the local optimum; (b) no matter clusters number is large (*k*>10) or small (*k*<3), it can be well adapted.

### Clustering solution-improved method

**Fig 5:**
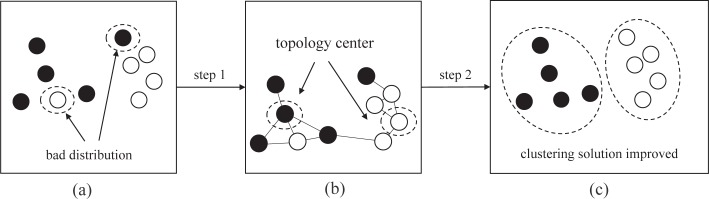
The process of solution-improved method. (a) Black points are one partition and the white is another. Obviously, there is a bad clustering solution. (b) Topology centers are found in Gabriel graph. (c) Improved solution is generated by agglomerative clustering method.

As a search-based method, PSO methods are easy to suffer from the curse of dimensionality with the increase of clustering dataset. As a result, the PSO technology is only to get a rough clustering distribution in the search process, an example is shown in Fig 5 (A).The main reason is that the PSO does not use any prior knowledge in the search process. To address this issue, we hope to find some properties of clustering to improve the solutions. In clustering problem, it is obvious that the probability of assigning to same group is higher where the data points are more similar. This implies that we can use this feature to improve the rough solution obtained by PSO. Fig 5 illustrate the solution-improved method, the steps are as follows.

*Step 1* Each partition has a topological center, and the centers can be found by Gabriel graph. First, calculate the *C* value of each partition in the clustering solution. *C* value is the number of data point’s neighbors that are of the same cluster in Gabriel graph. Then, the data with the largest *C* value is the topology center. If there are more than one topology center, take it randomly.

*Step 2* For each topology center, agglomerative clustering is used. It starts from *k* clusters, each containing one topology center and the other data points need to be reallocated. A series of nested merging is performed until all the data points are grouped into the *k* clusters. The algorithm processes the Gabriel graph between the *N* data points, and agglomerates according to their similarity or distance. Agglomerative clustering is based on a local connectivity criterion. The run time is *O*(*N*^2^).

*Step 3* The optimized clusters are converted into new particle vector. The *k* is placed in the first element of vector, and then the grouping number corresponding to each data points are divided by *k* and place them to other *N* elements. The converted particle vector is similar to *gbest* and *pbest* in Fig 2.

**Fig 6:**
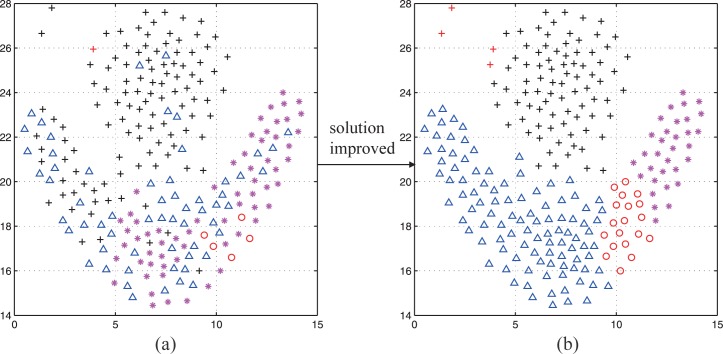
An example of clustering solution-improved method. (a) The original clustering distribution. (b) The improved clustering distribution.

The data points are rearranged to consistent with the characteristics of clustering by this technology, while the previous rough distribution is kept as much as possible. In this way, a better clustering solution can be obtained with acceptable computational cost even if the curse of dimensionality exists. It enhance the convergence and quality of solution greatly. An example is shown in Fig 6.

**Fig 7:**
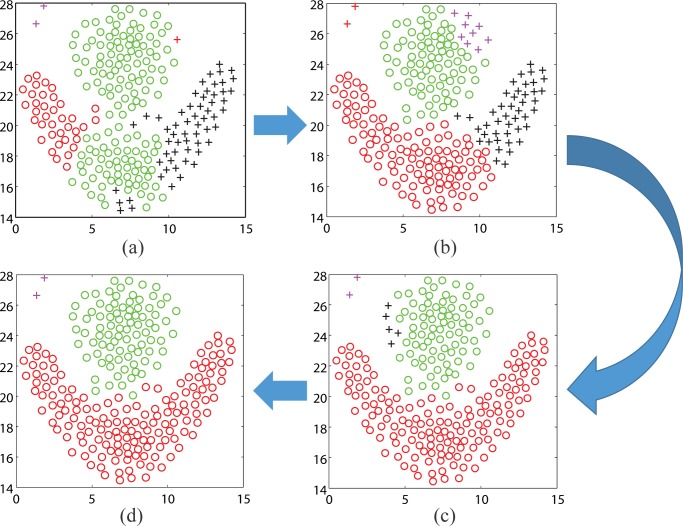
The change of *gbest* in a real search process.

In the actual search process, the rough partitioning of the clustering problem is obtained by PSO firstly. Then, the solution-improved method is used to get exact partitioning based on the rough solution. It must be emphasized that the improved solutions will help PSO to get better partitioning in the updating of particles. A real search process is shown in the Fig 7.

### Decision making

Similar to the leader particle selection, decision making is also an important issue when faced with many Pareto optimal solutions. In general, a decision maker is required to make a trade-off while deciding which solution to choose in presence of a large finite number of Pareto set. Here, we use the filter proposed in [42], which discards the solutions that are in the boundaries of the Pareto set. Based on that, decision maker finds the suitable solution based on crowding density in the remaining solution.

The use of the filter is controlled by two angles α_1_, α_2_, each controlling a ray passing through the origin (-*Mdc* is negative and a small value is used as the coordinate of the origin). Each region is described by one angle α_*i*_ in regard to the corresponding objective axis and the Pareto set belonging to the region is discarded. The angle α_*i*_ is updated in every iteration, and a quality evaluation function is used (please refer to [42] for details).The method can filter the Pareto set that barely take into account more than a single objective.

**Fig 8:**
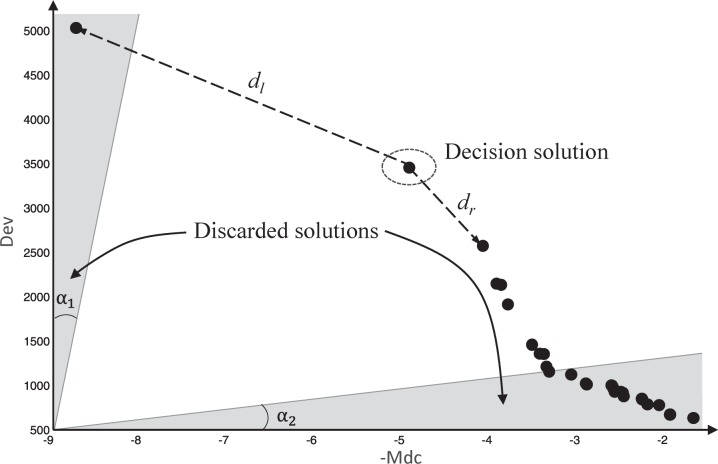
The process of making decision. At first, the Pareto sets of grey areas are discarded by filter. Then the decision solution of rest Pareto sets has the largest *sc*.

However, it is hard to make decision in the remaining Pareto set because they all have well trade-off between the two objective functions. According to the analysis of section 3.3.1, sparser Pareto solution has better *k* which makes the clusters well-separated. Therefore, we use the sparse coefficient to choose the sparsest particle among the remaining Pareto set. The sparse coefficient is defined as follows after all the Pareto solutions are normalized.

$${sc}_{i}=(d_{l,i}+d_{r,i})/2$$ (11)

where *sc*_*i*_ is the sparse coefficient of *i*th Pareto set, *d*_*l*,*i*_ and *d*_*r*,*i*_ are the Euclidean distance to nearest left Pareto solution and nearest right Pareto set, respectively. The Pareto solution with the largest sparse coefficient is taken as the decision solution, as shown in Fig 8.

In summary, the pseudo-code of IMCPSO presented in this paper is as follows.

**Algorithm 1** IMCPSO

Let *iter*_max_ be the max iteration times

Let *NonDom* be the reposition of non-dominated set

Calculate the similarity matrix and Gabriel graph of dataset

Initialize *swarm* randomly // using particle represent

Improve every *particle* in *swarm* //using clustering solution-improved method

**for** each *particle* in *swarm* **do**

        Evaluate objective functions for *particle*

**end for**

Store non-dominated *particles* in *NonDom*

**for** *iter* in 1 to *iter*_max_ **do**

        Select *gbest* in *NonDom* //using leader selection strategy

        Update *particle*, *pbest*

        **for** each *paricle* in *swarm* **do**

                Improve *particle* solution //using clustering solution-improved method

                Evaluate objective functions for *particle*

        **end for**

        Store non-dominated *particles* in *NonDom*

**end for**

Select decision solution in *NonDom* //using decision-making method

## Experiments and discussion

This section analyzes the performance of the IMCPSO. Firstly, 14 real-world datasets and 14 artificial datasets are analyzed. Secondly, the parameters in IMCPSO are configured and the results are calculated in each dataset. Finally, IMCPSO is compared with eight state-of-the-art single-objective algorithms (four algorithms need clusters number *k* and others do not), and one state-of-the-art multi-objective clustering algorithm. The above experiments are run on the MATLAB R2014a, windows 7(x64), Core i5 (2.4G Hz), 4 GB RAM.

### Datasets and comparison metrics

**Fig 9:**
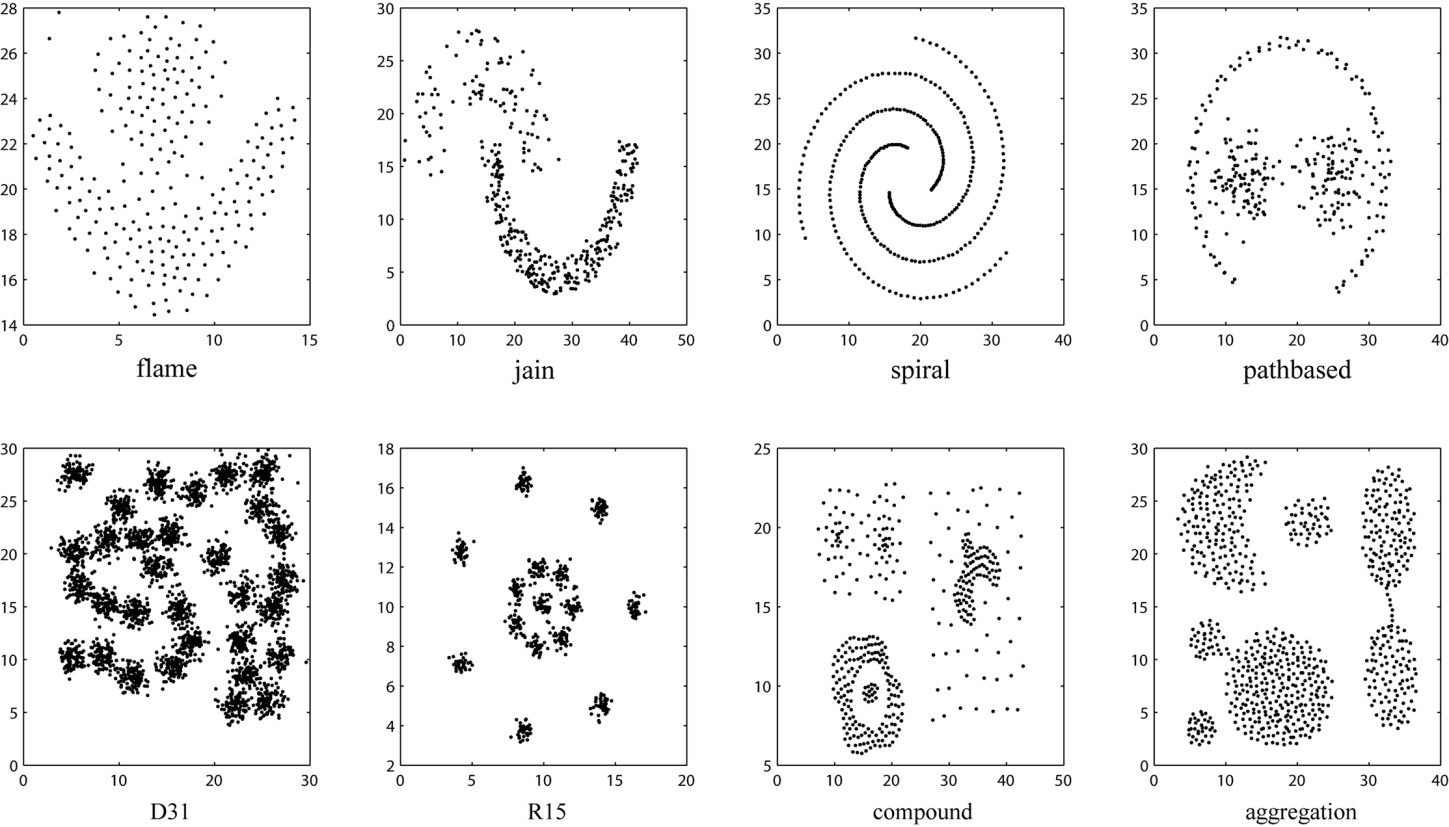
Example of 2-dimensional datasets with different shapes.

There are 28 datasets used in the experiments, where 14 datasets are artificial datasets and the other 14 datasets are real-world datasets, which collected from the UCI Machine Learning Repository[43] and KEEL[44]. The summary of datasets is shown in Table 1 and Fig 9 shows the shape of all 2-dimension datasets. The compressed file package of all datasets used in this paper is S1 File.

**Table 1:** Summary of the 14 real-world datasets (left block) and 14 artificial datasets (right block). The columns of each block are referred to the dimension of dataset (*D*), to the number of classes (*K*) and to the number of instances (*N*).

Dataset	*D*	*K*	*N*	Dataset	*D*	*K*	*N*
flame	2	2	240	appendicitis	7	2	106
jain	2	2	373	dermatology	34	6	358
spiral	2	3	312	ecoli	7	8	336
pathbased	2	3	299	glass	9	7	214
D31	2	31	3100	haberman	3	2	306
R15	2	15	600	housevotes	16	2	232
compound	2	6	399	ionosphere	33	2	351
aggregation	2	6	788	iris	4	3	150
dim032	32	16	1024	segment	19	7	2310
dim064	64	16	1024	vehicle	18	4	846
dim128	128	16	1024	wdbc	30	2	569
dim256	256	16	1024	wine	13	3	178
dim512	512	16	1024	wisconsin	9	4	683
dim1024	1024	16	1024	zoo	16	7	101

The accuracy of each solution was quantified using the Adjusted Rand index (ARI)[45] since the standard labels exist in each dataset. ARI measures the similarity between the obtained clusters and the true ones and lies in the interval [–1,1]. This index has high value when the obtained clusters are more similar to the true ones.

### Configuration

The parameters of the proposed algorithm are similar to other multi-objective PSO, including inertia weight *w*, learning parameters *c*_1_ and *c*_2_, maximum iteration number *iter*_max_, number of particle *N*_*p*_ and maximum number of clusters *k*_max_. We carried out 20 independent experiments with the different setting in five datasets and got the best configuration. The parameters are set as follows: *w* = 0.85, *c*_1_ = *c*_2_ = 0.7, *iter*_max_ = 500, *N*_*p*_ = 20 and *k*_max_ = 20.

### Experimental comparisons

The experimental comparison is divided into three parts. First, we compare it with the single-objective clustering algorithms that can determine *k* automatically. Secondly, we compare it with the single-objective clustering algorithms that needs *k* in advance, the correct *k* is provided for these algorithms to ensure the best performance. Finally, IMCPSO compares with the state-of-the-art multi-objective clustering algorithm.

#### Comparison with automatic k-determination clustering methods

There are mainly four clustering algorithms with automatic *k*-determination: AP clustering, Mean Shift, Ward and DBSCAN. After multiple runs on datasets, the outputs of above algorithms are compared with IMCPSO as shown in the Table 2, and the best result among five methods is shown in bold.

**Table 2:** Mean of ARI on the outputs of IMCPSO (over 40 independent runs), AP clustering, Mean Shift, Ward and DBSCAN.

datasets type	datasets	IMCPSO	AP clustering	Mean Shift	Ward	DBSCAN
shape cluster datasets	Flame	**0.92**	0.44	0.52	0.29	0.03
	Jain	0.72	0.51	0.17	0.57	**0.89**
	Spiral	**0.98**	0.01	0.06	0.13	0.43
	Pathbased	**0.89**	0.40	0.40	0.46	0.66
	D31	0.75	0.28	0.06	**0.91**	0.01
	R15	**0.96**	0.19	0.24	0.94	0.93
	Compound	**0.82**	0.46	0.72	0.50	0.81
	Aggregation	**0.86**	0.68	0.62	0.70	0.33
high dimension datasets	Dim032	**0.99**	0.95	0.01	0.96	0.12
	Dim064	0.96	**0.96**	0.01	0.96	0.15
	Dim128	0.86	**0.98**	0.01	0.96	0.15
	Dim256	**0.96**	0.95	0.01	0.95	0.15
	Dim512	0.89	0.96	0.01	**0.98**	0.15
	Dim1024	0.92	**0.98**	0.01	0.96	0.15
real-world datasets	Appendicitis	**0.37**	0.12	0.31	0.31	0.01
	Dermatology	0.31	0.57	0.06	**0.93**	0.01
	Ecoli	**0.46**	0.36	0.04	0.34	0.02
	Glass	**0.31**	0.22	0.24	0.26	0.03
	Haberman	**0.18**	0.03	0.07	0.05	0.01
	Housevotes	0.33	0.08	0.01	**0.61**	0.11
	Ionosphere	**0.30**	0.18	0.01	0.18	0.01
	Iris	**0.88**	0.57	0.57	0.53	0.01
	Segment	**0.47**	0.35	0.01	0.44	0.01
	Vehicle	**0.16**	0.10	0.01	0.09	0.02
	Wdbc	0.30	0.18	0.01	**0.36**	0.01
	Wine	0.39	0.58	-0.01	**0.75**	0.01
	Wisconsin	0.45	0.16	**0.76**	0.74	-0.07
	Zoo	**0.66**	0.41	0.03	0.64	-0.02

#### Comparison with k-needed clustering methods

The performance of IMCPSO has been compared with those K-means, spectral clustering, average-link and Birch, as those algorithms have in common is that require the clusters number *k* in advance. The correct *k* is fed into these algorithms to ensure the best performance while IMCPSO does not need *k*. The results of comparison are shown in Table 3, and the best result among five methods is shown in bold.

**Table 3:** Mean of ARI measured on the outputs of IMCPSO (over 40 independent runs), K-means, spectral clustering, average-link and Birch.

datasets type	datasets	IMCPSO	K-means	Spectral clustering	Average-link	Birch
shape cluster datasets	Flame	**0.92**	0.48	0.26	0.01	0.49
	Jain	0.72	0.51	0.72	0.02	**0.92**
	Spiral	**0.98**	0.01	0.46	0.49	0.01
	Pathbased	**0.89**	0.48	0.50	0.39	0.47
	D31	0.75	0.87	**0.94**	0.14	0.49
	R15	**0.96**	0.93	0.82	0.48	0.58
	Compound	**0.82**	0.50	0.49	0.58	0.78
	Aggregation	**0.86**	0.64	0.78	0.81	0.75
high dimension datasets	Dim032	**0.99**	0.99	0.09	0.92	0.99
	Dim064	0.96	**0.99**	0.12	0.89	0.99
	Dim128	0.86	**0.99**	0.08	0.83	0.99
	Dim256	0.96	**0.99**	0.07	0.80	0.99
	Dim512	0.89	**0.99**	0.10	0.79	0.99
	Dim1024	0.92	**0.99**	0.08	0.72	0.99
real-world datasets	Appendicitis	0.37	0.39	**0.46**	0.14	0.42
	Dermatology	0.31	0.72	**0.91**	0.2	0.92
	Ecoli	**0.46**	0.31	0.37	0.05	0.45
	Glass	**0.31**	0.24	0.17	0.02	0.26
	Haberman	**0.18**	0.02	-0.01	0.01	0.01
	Housevotes	0.33	0.61	**0.64**	0.01	0.61
	Ionosphere	**0.30**	0.15	-0.03	0.01	0.15
	Iris	**0.88**	0.57	0.43	0.54	0.54
	Segment	**0.47**	0.46	0.3	0.02	0.3
	Vehicle	**0.16**	0.08	0.08	0.01	0.09
	Wdbc	0.30	0.69	**0.77**	0.01	0.51
	Wine	0.39	0.77	**0.83**	0.02	0.71
	Wisconsin	0.45	**0.75**	0.52	0.02	0.73
	Zoo	0.66	**0.71**	0.49	0.65	0.8

#### Comparison with the multi-objective clustering method

The state-of-the-art multi-objective clustering algorithm is the MCPSO proposed in [20]. The ARI index of same datasets is compared since the author does not provide the source code of MCPSO, and results are shown in Table 4, the best result among two methods is shown in bold. In particular, the maximum number of iterations *iter*_max_ is 1000 in MCPSO, while *iter*_max_ is only 500 in IMCPSO.

**Table 4:** Mean of ARI measured on the outputs of IMCPSO and MCPSO (over 40 independent runs).

datasets	IMCPSO	MCPSO
Flame	**0.92**	0.73
Jain	**0.72**	0.66
Spiral	**0.98**	0.82
Pathbased	**0.89**	0.81
R15	**0.96**	0.88
Compound	0.82	**0.86**
Aggregation	0.86	**0.87**
Dim032	**0.99**	0.83
Dim064	**0.96**	0.85
Dim128	**0.86**	0.80
Dim256	**0.96**	0.72
Dim512	**0.89**	0.71
Glass	0.31	**0.88**
Wdbc	0.30	**0.79**

### Results analysis

In the shape clustering datasets, it can be seen that baseline clustering methods have better performance than IMCPSO on only 2 datasets (Jain and D31) in terms of ARI index, because the IMCPSO shows a good performance dealing with arbitrary clusters. It is worth noted that clusters number of D31 is 31, but the *k*_max_ in configuration of IMCPSO is 20, this is the reason why the IMCPSO shows bad performance on this dataset.

The high dimension datasets are spherical Gaussian distribution, which can be assumed as the most suitable for the AP clustering, Ward, Birch and K-means. In particular, K-means and Birch with correct *k* shows high performance on those datasets. Nevertheless, the performance of IMCPSO is still very well and overall ARI index is above 0.9. In addition, the IMCPSO has best performance on 2 datasets (Dim032 and Dim256).

In the real-world datasets, the performance of *k*- needed clustering methods are better than the automatic *k*-determination methods since the correct *k* is given. Nevertheless, the performance of IMCPSO is better than the *k*-needed clustering algorithms in 7 datasets (Ecoli, Glass, Haberman, Ionosphere, Iris, Segment, Vehicle). Moreover, the performance of IMCPSO is better than the state-of-the-art multi-objective method (MCPSO) in 10 datasets of total 14 datasets with less iteration number.

To examine the performance in terms of the statistical differences between the proposed algorithm and other contrastive algorithms, we performed the Wilcoxon Signed-Rank test [46] on the mean ARI results of each datasets type. The Wilcoxon signed-rank test is a non-parametric statistical hypothesis test and it is used to determine differences between groups of paired data. Table 5 is listed with the results of signed-ranks test between IMCPSO and contrastive algorithms, *N* is the number of data and the statistically significant results are highlighted in bold text.

**Table 5:** The results of Wilcoxon signed-rank test of IMCPSO with other contrastive algorithms.

Contrastive methods	Datasets type	*N*	Sum of ranks	*p* value	Contrastive methods	Datasets type	*N*	Sum of ranks	*p* value
AP clustering	shape	8	36	**0.008**	Mean Shift	shape	8	36	**0.008**
	high dimension	6	3	0.313		high dimension	6	21	**0.031**
	real-world	14	85	**0.042**		real-world	14	96.5	**0.003**
Ward	shape	8	32.5	**0.047**	DBSCAN	shape	8	33	**0.039**
	high dimension	6	3	0.313		high dimension	6	21	**0.031**
	real-world	14	52.5	0.998		real-world	14	105	**0.001**
K-means	shape	8	34	**0.023**	Spectral clustering	shape	8	25	0.078
	high dimension	6	0	0.063		high dimension	6	21	**0.031**
	real-world	14	42	0.531		real-world	14	53.5	0.964
Average-link	shape	8	36	**0.008**	Birch	shape	8	33	**0.039**
	high dimension	6	21	**0.031**		high dimension	6	0	0.063
	real-world	14	105	**0.001**		real-world	14	41.5	0.510
MCPSO	shape	7	3	0.078					
	high dimension & real-world	7	15	0.937					

For shape datasets, the differences between IMCPSO and other 7 methods are significant with *P*<0.05(AP clustering, Mean Shift, Ward, DBSCAN, K-means, Average-link and Birch). For high dimension datasets, there are 4 methods have significant differences between IMCPSO and themselves (Mean Shift, DBSCAN, Average-link and Spectral clustering). There are 4 contrastive methods (AP clustering, Mean Shift, DBSCAN and Average-link) have significant differences to IMCPSO in the real-world datasets. It is worth noted that no significant difference between IMCPSO and MCPSO, but the *p* value is very small (0.078) in the shape datasets.

In summary, the performance of IMCPSO is superior to the other clustering methods in the real-world and shape datasets. Especially, it has significant differences to most contrastive algorithms in the shape datasets. In the high dimension datasets, the differences between IMCPSO and other best algorithms are small, while the IMCPSO has less iteration and does not need *k*.

## Conclusion

In this study, a multi-objective clustering method (i.e., IMCPSO) is proposed. Particle representation, leader selection and decision maker are taken into consideration, and clustering is made automatically. The IMCPSO extends the multi-objective PSO to clustering problem, which greatly increases the efficiency in searching clustering solution and helps the algorithm to avoid trapping in local optimum. The performance of IMCPSO has been studied in comparison with eight single-objective clustering methods and a state-of-the-art multi-objective clustering algorithm. The results, obtained over 28 benchmark datasets, show that the IMCPSO is able to outperform the other algorithms in term of accuracy over the majority of datasets. Moreover, IMCPSO does not need to provide the number of clusters in advance.

In our future research, more objective functions will be investigated, and we plan to enhance the performance of IMCPSO to solve the real-world clustering problem. We have already begun with it and found it is efficient in spatial clustering problems.

## Supporting information

S1 File The compressed file package of all datasets used in this paper. (ZIP)
